# Spatial clusters of suicide in Australia

**DOI:** 10.1186/1471-244X-12-86

**Published:** 2012-07-23

**Authors:** Xin Qi, Wenbiao Hu, Andrew Page, Shilu Tong

**Affiliations:** 1 Queensland University of Technology, Brisbane, Australia; 2 University of Queensland, Brisbane, Australia

## Abstract

**Background:**

Understanding the spatial distribution of suicide can inform the planning, implementation and evaluation of suicide prevention activity. This study explored spatial clusters of suicide in Australia, and investigated likely socio-demographic determinants of these clusters.

**Methods:**

National suicide and population data at a statistical local area (SLA) level were obtained from the Australian Bureau of Statistics for the period of 1999 to 2003. Standardised mortality ratios (SMR) were calculated at the SLA level, and Geographic Information System (GIS) techniques were applied to investigate the geographical distribution of suicides and detect clusters of high risk in Australia.

**Results:**

Male suicide incidence was relatively high in the northeast of Australia, and parts of the east coast, central and southeast inland, compared with the national average. Among the total male population and males aged 15 to 34, Mornington Shire had the whole or a part of primary high risk cluster for suicide, followed by the Bathurst-Melville area, one of the secondary clusters in the north coastal area of the Northern Territory. Other secondary clusters changed with the selection of cluster radius and age group. For males aged 35 to 54 years, only one cluster in the east of the country was identified. There was only one significant female suicide cluster near Melbourne while other SLAs had very few female suicide cases and were not identified as clusters. Male suicide clusters had a higher proportion of Indigenous population and lower median socio-economic index for area (SEIFA) than the national average, but their shapes changed with selection of maximum cluster radii setting.

**Conclusion:**

This study found high suicide risk clusters at the SLA level in Australia, which appeared to be associated with lower median socio-economic status and higher proportion of Indigenous population. Future suicide prevention programs should focus on these high risk areas.

## Background

Suicide remains an important public health problem in Australia, with approximately 2,000 suicide cases occurring nationally since the mid-1990s (an average annual incidence of approximately 9.7 per 100,000) [1,2]. Suicide in Australia has also been shown to vary by area-based socio-economic strata [3], by measures of urban–rural residence [4], and by small-area geographic units [5,6]. Geographic analyses to identify areas of high suicide risk have also been conducted in other countries, such as Belgium [7], Taiwan [8,9], United Kingdom [10-15], United States [16,17], and Brazil [18].

Spatial analysis methods have been used to identify high risk areas of suicide in previous studies [6,8-11,13,17,18]. However, some studies focused only on urban areas or discrete geographic areas (*e.g.*, state level) [17,18], and others used smoothed standardized mortality ratios (SMRs) to identify areas with high or low suicide incidences [9,10]. Such smoothing methods may be inadequate in areas with sparse population and very few suicide cases [9,10]. Studies in Brazil [18] and the United Kingdom [16] did not examine the pattern of suicide risk in rural and remote areas, which may substantially differ from urban areas. In Australia, a few reports and studies have examined the suicide pattern across the whole country [1,2,19], but the spatial pattern of suicide has not been investigated in the national wide to date. At the state level some studies on suicide patterns have also been implemented in New South Wales [20] the Northern Territory [21], and South Australia [22], but they did not use spatial analysis. A spatial analysis can identify areas with high suicide mortality, visualize the pattern of high risk areas and explore possible reasons for high risk, *e.g.* socioeconomic variety across different areas. Given the geographic and socio-demographic diversity across Australia, it is important to examine the suicide pattern in the whole of Australia. This study is based on our previous research which was conducted in Queensland [6].

## Methods

### Data sources

Suicide data (1999–2003), including sex, age, country of birth, year and month of suicide and statistical local area (SLA) code were provided by Australian Bureau of Statistics (ABS). Access to recent suicide mortality data was unavailable as the related procedure is currently under review. The institutional ethics approval was granted by the Human Research Ethics Committee, Queensland University of Technology.

In 2001, Australia was divided into 1,346 SLAs in eight states and territories: New South Wales (NSW), Victoria (VIC), Queensland (QLD), South Australia (SA), Western Australia (WA), Tasmania (TAS), Northern Territory (NT) and Australian Capital Territory (ACT). 2001 Census data (CDATA 2001) included information on digital statistical boundaries, base maps, and SLA information (name, code, area (km²), longitude and latitude of the centroids, and population by age and gender). Some suicide cases (less than 1% of total suicides) were excluded from this study due to a lack of documentation on the SLA code. Socio-demographic data, such as Socio-Economic Indexes for Area (SEIFA), Indigenous status and unemployment rate at the SLA level were obtained from CDATA 2001. These data were used as potential explanatory factors for clusters. A higher SEIFA score indicates higher socio-economic status at the SLA level.

### Data analysis

A series of statistical and Geographical Information System (GIS) methods were applied to analyse the data. GIS is a powerful tool to store, retrieve and display spatial data. The spatial locations and trends of disease outbreaks can be identified by GIS [23]. GIS can also spatially explore disease aetiologies, such as socio-environmental factors associated with disease [23]. Then GIS can help public health workers to design and implement effective disease control and prevention programs at a local level. Descriptive analysis was conducted to explore the characteristics of each variable, such as suicide cases, population and suicide age-adjusted standardised mortality (ASM) by gender [24]. GIS and mapping approaches were applied to investigate the spatial distribution of suicide SMR by gender at the SLA level [25]. A direct method, which was used in our previous Queensland study [6], was applied to calculate the ASM by gender for each SLA. Then the SMR was also calculated by using the mean of annual mortality of suicide in the whole Australia (1999–2003) as a reference, stratified by gender.

To identify high risk areas, we applied spatial cluster analysis to identify the randomly-distributed suicide cases and to explore primary clusters (*i.e.*, those with the highest risk among all clusters) and secondary clusters (other high risk clusters with significance), using SaTScan [26,27]. A Poisson regression model was performed to compute the mean relative risk (RR) of each cluster and likelihood ratio to identify the two types of clusters as circular windows. We also used maximum population size of cluster (covering less than 50%, 25% and 10% of total population) and maximum length of cluster radii (100 km and 400 km) to examine whether these factors could determine the pattern of clusters, as 100 km radii may cover most of urban areas and 400 km radii may cover most of rural or remote areas. Different population size and radii of cluster can be selected in SaTScan. As some documents indicated that most of suicide cases were aged between 15 and 54 [1,2], the spatial clusters of suicide for this age group were specifically examined as two groups (15 to 34-year and 35 to 54-year). The median SEIFA and mean values of other socio-demographic factors of clusters were calculated and compared with the national average, in order to identify whether there were any differences in these factors between clusters and other areas.

## Results

11,586 suicide deaths were included in the analysis covering the whole study period, with 9,142 males and 2,444 females (male/female ratio: 3.74). 4,472 (38.6%) of total suicides were aged between 15 and 34 (3,577 males and 895 females) and 4,540 (39.2% of total) between 35 and 54 (3,579 males and 961 females). The metropolitan areas of capital cities covered only 0.5% of the total area examined but contained 63.4% of total population [28] and 59.4% of total suicide deaths. Rural and remote SLAs had sparse population density and many had no suicide cases during the study period. Table 1 indicates the distribution of area, population and suicide by SLA. All the variables differed substantially across SLAs.

**Table 1:** Statistical summary of variables at the SLA level (1,346 SLAs)

	**Mean**	SD^*^	**Minimum**	**Percentile**			**Maximum**
				**25**	**50**	**75**	
Area (km^2^)	5735.93	28213.706	0.33	7.15	87.81	2209.99	671465.75
Male population	6966.90	11235.696	0	1423	3011	7200	90716
Female population	7150.07	11666.613	0	1389	2984	7329	91399
Population density (per km^2^)	730.28	1074.785	0	2.00	132.32	1355.72	10448.12
Suicide cases	8.63	13.389	0	1	4	10	123
Male suicide cases	6.81	10.539	0	1	3	8	102
Female suicide cases	1.82	3.191	0	0	1	2	26
Male SMR	1.12	1.240	0	0.53	0.97	1.49	25.24
Female SMR	1.98	1.882	0	0	0.44	1.35	34.20
Proportion of male population aged 15 to 34 (%)	27.19	7.357	0	22.67	26.70	30.48	85.00
Proportion of male population aged 35 to 54 (%)	29.10	5.038	0	27.33	29.02	30.90	100.00
Proportion of male suicide aged 15 to 34 (%)	32.18	30.686	0	0	33.33	50.00	100.00
Proportion of male suicide aged 35 to 54 (%)	31.80	29.859	0	0	33.33	50.00	100.00
Proportion of female population aged 15 to 34 (%)	26.60	7.094	0	22.02	25.80	29.84	86.01
Proportion of female population aged 35 to 54 (%)	29.03	4.990	0	26.84	28.85	31.34	100.00
Proportion of female suicide aged 15 to 34 (%)	18.46	31.960	0	0	0	33.33	100.00
Proportion of female suicide aged 35 to 54 (%)	22.31	34.448	0	0	0	40.00	100.00

SD: Standard deviation.

**Figure 1:**
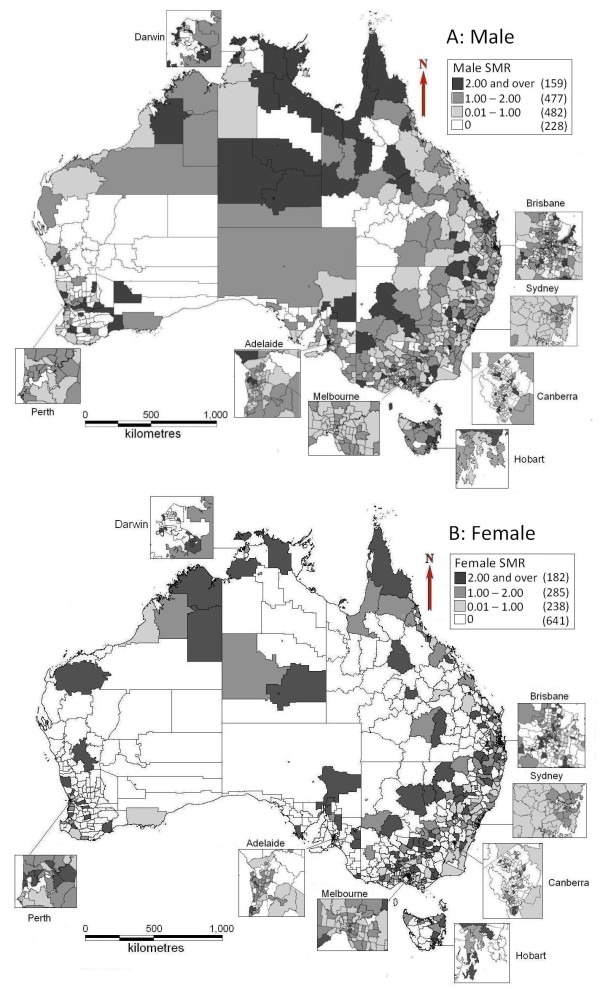
Age-adjusted standardised mortality ratio of suicide at the SLA level by gender.

Figure 1A indicates that the central south of Northern Territory (NT), north, inland and the eastern coast of Queensland (QLD), inland areas of New South Wales (NSW) and Western Australia (WA), and the eastern coast of Tasmania (TAS) had higher male SMRs than other areas. There were no male suicide cases during the study period in central and some southern areas of WA; central, north and southwest QLD; and northwest NSW. North and inland areas QLD, some areas in NSW and VIC, north and some western areas in WA, and eastern TAS had higher female suicide SMRs than other areas (Figure 1B). However, 47.6% of SLAs had no female suicide cases during the study period.

**Figure 2:**
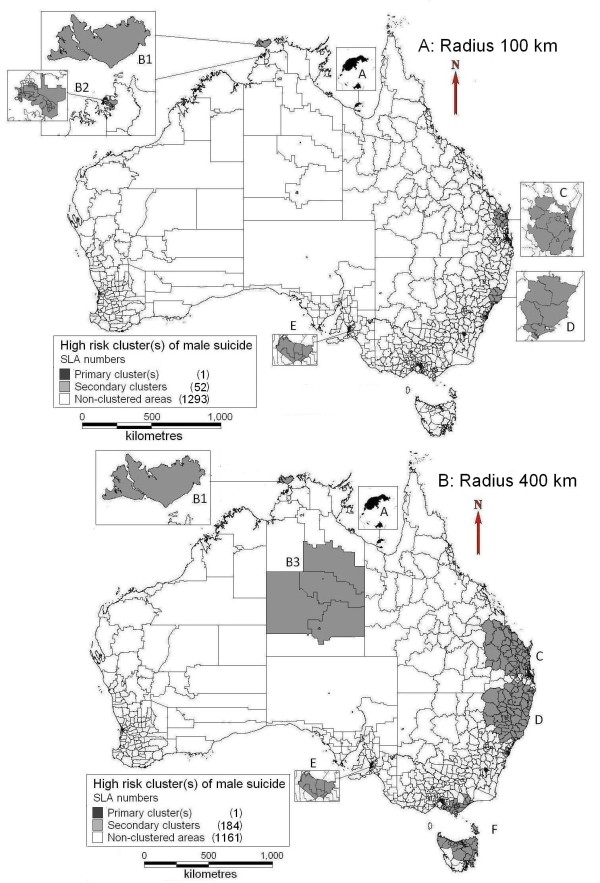
Clusters of male suicide (A: radius limit of 100 km; B: radius limit of 400 km).

Figure 2 shows the spatial clusters of male suicide. Mornington Shine (A in Figure 2A and 2B) in the northwest of QLD was identified as the primary cluster. Bathurst-Melville area (B1, north of NT) and some suburbs of Adelaide in SA (E) were discovered as secondary clusters at both of the maximum radii of 100 km (Figure 2A) and 400 km (Figure 2B). Other secondary clusters changed with selection of different maximum lengths of cluster radii. All the detailed information of male suicide clusters at different cluster radii is provided in Table 2. For female suicide clusters, only one primary cluster was identified near Melbourne after adjustment for radius and age group (Table 2). We also tested the impact of different population size (*i.e.*, clusters of less than 50%, 25%, 10% and 5% of total population) on the spatial pattern of suicide but did not find any significant difference.

**Table 2:** Information on clusters of high risk at the SLA level

**Type**	**Radius (km)**	**Cluster number in figures**	**SLA number(s) and location**	**Cluster radius (km)**	**Area (km**^**2**^**)**	**Population**	**Number of cases**	**Expected cases**	**RR**	**95% CI**	**P value**
**Primary** (male)	100 km/400 km	A	1(Mornington Shire, northwest of QLD)	0	1,231.25	397	12	0.45	26.49	15.07, 45.95	<0.001
Secondary (male)	100 km	B1	1(Bathurst-Melville, north of NT)	0	7,492.01	873	13	0.98	13.25	7.59, 22.69	<0.001
		B2	24 (Darwin, NT)	10.58	250.90	26,077	58	30.88	1.88	1.50, 2.51	0.037
		C	13 (East of QLD)	80.45	15,917.11	68,830	128	77.52	1.66	1.37, 1.98	<0.001
		D	8 (Central Coast areas, NSW)	66.45	13,740.91	140,699	222	160.32	1.39	1.21, 1.58	0.012
		E	6 (Adelaide, SA)	5.32	95.57	51,536	99	60.51	1.64	1.38, 2.05	0.019
	400 km	B1	1(Bathurst-Melville, north of NT)	0	7,492.01	873	13	0.98	13.25	7.59, 22.69	<0.001
		B3	6 (Inland of NT)	367.33	661,409.44	8,765	30	9.81	3.07	2.09, 4.28	0.0012
		C	66 (East of QLD)	272.48	157,690.60	309,537	461	349.24	1.34	1.19, 1.44	<0.001
		D	58 (Northeast NSW & south of QLD)	261.50	180,322.20	388,923	559	438.63	1.29	1.16, 1.38	<0.001
		E	6 (Adelaide, SA)	5.32	95.57	51,536	99	60.51	1.64	1.38, 2.05	0.026
		F	47 (Southeast VIC & north of TAS)	284.63	64,048.26	185,052	277	209.16	1.33	1.16, 1.48	0.027
**Primary** (female)	100 km/400 km		7 (Melbourne area, VIC)	6.00	88.46	154,518	74	39.33	1.91	1.52, 2.41	0.001

**Figure 3:**
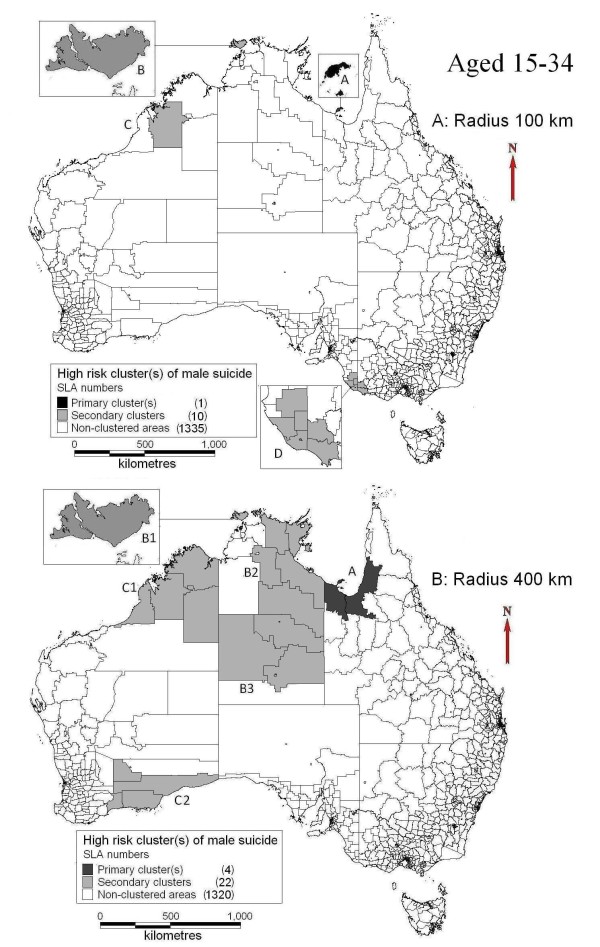
Clusters of male suicide (aged 15–34. A: radius limit of 100 km; B: radius limit of 400 km).

**Figure 4:**
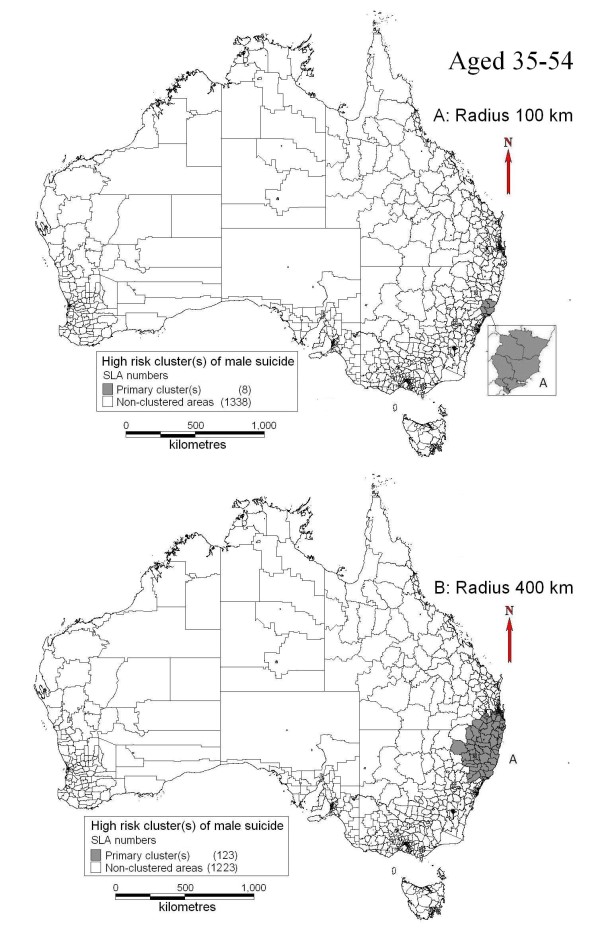
Clusters of male suicide (aged 35–54. A: radius limit of 100 km; B: radius limit of 400 km).

The clusters of male suicide in different age groups were also presented. Mornington Shire was the primary cluster of male suicide for youths (15 to 34-year) at the maximum radii of 100 km (Figure 3A) and this cluster expanded to cover much larger areas of Northwest QLD (including Mornington Shire) at the maximum radii of 400 km (Figure 3B). Bathurst-Melville area was identified as a secondary cluster with different cluster radii selection (Figure 3A and 3B). Other secondary clusters changed with the selection of different maximum lengths of cluster radii (Figure 3A and 3B). For suicide aged between 35 and 54, only one cluster was found in the Central Coast of NSW at the maximum radii of 100 km (Figure 4A) and this cluster expanded to cover the whole Northeast NSW and parts of South QLD at the maximum radii of 400 km (Figure 4B). Table 3 provides detailed information of clusters at different age groups and cluster radii.

**Table 3:** Information on clusters of high risk (male suicide among 15 to 34 years and 35 to 44 years) at the SLA level

**Type**	**Radii (km)**	**Cluster number in figures**	**SLA number(s) and location**	**Cluster radii (km)**	**Area (km**^**2**^**)**	**Population**	**Number of cases**	**Expected cases**	**RR**	**95% CI**	**P value**
**Primary** (15 to 34-year)	100	A	1(Mornington Shire, Northwest of QLD)	0	1,231.25	184	10	0.25	40.32	22.05, 73.72	<0.001
	400	A	4(Northwest of QLD)	211.91	111,527.17	1258	19	1.70	11.23	7.18, 17.56	<0.001
Secondary (15 to 34-year)	100	B	1(Bathurst-Melville, north of NT)	0	7,492.01	431	12	0.58	20.66	11.82, 36.13	<0.001
		C	1 (North of WA)	0	104,079.96	1,889	13	2.55	5.11	2.97, 8.77	0.013
		D	8 (Southwest of VIC & southeast of SA)	94.98	16,580.74	9,003	30	12.17	2.48	1.73, 3.55	0.044
	400	B1	1(Bathurst-Melville, north of NT)	0	7,492.01	431	12	0.58	20.66	11.82, 36.13	<0.001
		B2	7 (Northeast, NT)	381.84	263,138.40	4,431	24	5.99	4.03	2.70, 6.01	<0.001
		B3	6 (Inland of NT)	367.33	661,409.44	3,601	24	4.87	4.96	3.32, 7.40	<0.001
		C1	4 (Northeast, WA)	314.37	420,420.00	6,693	31	9.05	3.45	2.42, 4.91	<0.001
		C2	5 (Southeast, WA)	296.51	179,497.34	7,994	30	10.81	2.79	1.95, 4.00	0.008
**Primary** (35 to 54-year)	100	A	8(Central Coast areas, NSW)	66.45	13,740.91	45,405	98	60.56	1.64	1.34, 2.00	0.021
	400	A	123(Northeast NSW & south of QLD)	303.60	163,267.65	235,694	420	314.36	1.38	1.25, 1.53	<0.001

Table 4 shows the socio-demographic characteristics of high risk clusters for male suicide at all ages. Primary and secondary clusters had a higher proportion of Indigenous population than the national average level (about 39 times higher in the primary cluster; secondary clusters: 41% higher in the 100 km radii and 73% higher in the 400 km radii settings). The median SEIFA scores in the clusters were lower than the national median SEIFA score (Table 4), indicating that high risk clusters had lower socioeconomic status than the national average level. For female suicide, we did not find any significant difference in socio-demographic factors between cluster areas and other areas.

**Table 4:** Socio-demographic factors in cluster areas (all male suicide)

**Type**	**Radius (km)**	**Proportion of Indigenous population (%)**	**Unemployment rate (%)**	**SEIFA (median)**
Primary cluster		86.50	1.17	882.03
Secondary clusters	100 km	3.07	8.70	827.51
	400 km	3.58	5.51	910.11
National level		2.17	6.26	1000

## Discussion

This study explored the geographical distribution of suicide and clusters of high risk in Australia. There was a higher risk of male suicide in the north of QLD, some areas in the east coast of QLD and TAS, inland areas in QLD, NSW and WA, and central south areas in NT (SMR > 2), compared with the national average. Female suicide incidence was significantly lower than male suicide incidence overall, and over 40% of all SLAs had no female suicide deaths in the study period. Only one cluster of female suicide was identified in this study.

In capital cities, the numbers of suicide cases in each year were relatively steady. Only some SLAs near Adelaide and Darwin were identified as clusters of high risk of male suicide. The Darwin metropolitan area has a smaller population than other capital cities, as well as a smaller average population at the SLA level. Thus the suicide mortality was high in some SLAs in Darwin. A report in South Australia indicated that the west of Adelaide, where the cluster in this study lies, had higher incidence of mental and behavioural disorders than that of the whole of Adelaide and Australia, which may be associated with suicidal behaviours [29]. For female suicide, many SLAs with high SMR had only a small number of suicide cases. Thus the clusters of female suicide were not as obvious as male suicide. For example, Moreton Island near Brisbane had a SMR of 22.2 compared with the national female suicide incidence, but had only 1 female suicide. Thus these SLAs were not identified as high risk clusters by SaTScan. This phenomenon can also be found in some SLAs having a high male suicide SMR but with a very small population size (*e.g.*, Fyshwick in Canberra).

In the spatial cluster analysis of total and 15 to 34-year male suicide, the Mornington Shire was identified as the whole or part of a primary cluster of high risk, as well as the secondary cluster of Bathurst-Melville area in both when setting the maximum radii at 100 km and 400 km. Our previous studies indicated that suicide was higher in the areas with larger proportion of Indigenous population than other areas and these areas usually had lower socioeconomic status [5,6]. The Bathurst-Melville area also had a low SEIFA score and over 80% of local population were Indigenous. The findings in previous and current studies are similar which suggests that the same set of determinants of suicide clusters exist at both national and state levels. Additionally, social disruption and alcohol abuse may also contribute to the high suicide incidence, according to some studies [30,31] and media reports [32-34] relating to the Mornington Shire and Bathurst-Melville. Due to very small numbers of 35 to 54-year suicides in Mornington Shire (2 cases) and Bathurst-Melville (1 case), these two areas were not identified as high risk clusters in this age group.

The sizes and positions of other secondary clusters changed with the selection of different radius and age structure. A cluster with a large radius limit could hide the information of smaller areas within the cluster, while the selection of clusters with a small radius limit may miss some significant high risk areas compared with a larger radius. SaTScan applies a circling approach to select all the geographical units in one place (*e.g.*, northeast of NSW) as a particular cluster, in which these areas may be heterogeneous, especially when the radius setting is large [35,36]. Thus some clusters tend to include both high risk areas and adjacent areas with low risk or even no suicide cases. Compared with the study in QLD [6], this study covered a much larger area, thus SaTScan could select more SLAs within one cluster (larger radius). This can explain that the cluster (400 km of maximum radius setting) in the east of QLD in this national study had 13 times the size (km^2^), 9 times of population and 7 times the number of suicide cases of that in the QLD study [6]. There are some variations of significant clusters (especially secondary) between males at different age groups, due partly to the different distribution of age groups of population and suicides across SLAs (Table 1).

There are several strengths in this study. This is the first study to examine the spatial clusters of suicide at a national level in Australia. Clustered areas of high risk need to be identified to facilitate the assessment of factors associated with high suicide risk and to design effective public health interventions. This study explored the variations of spatial clusters at different settings, including various cluster radii and age groups. Finally, the method developed in this study may contribute to identifying high risk areas of other mental health problems or diseases and improving mental health promotion.

The limitations of this study should also be acknowledged. Firstly, the data are not current and covered a period when Australian suicide was declining after a peak in 1997. Thus it may not represent the current patterns of suicide, potentially limiting its use in current suicide prevention strategies. Secondly, detailed personal information (*e.g.*, health status before death and suicide methods) was not available in this study. Thus it is difficult to assess how suicidal behaviours may be modified by individual-level factors in these areas. Finally, some risk factors such as drug and alcohol use, and potential modifying factors such as suicide prevention activities and the provision of healthcare services were not taken into account. These data are not routinely collected at the SLA level, but it is likely that such factors would affect spatial patterns of suicide.

Based on the findings of this study, some recommendations can be proposed. Firstly, even though most suicides occur in capital cities due to a large urban population, some rural and remote areas had high suicide risk, which is consistent with previous studies of rural suicide in Australia [3-6]. It is necessary to collect more detailed information (*e.g.*, suicide methods, mental health status of suicide cases and general population) in high risk areas, to discern the causes of suicide and to help design specific suicide prevention activities in these areas. Previous studies have found that a series of suicide control and prevention activities targeting the general population, such as antidepressant use [37,38], firearm and pesticide restrictions [39,40], domestic gas detoxification [41,42], primary care physician education [43,44], and public education campaigns [45,46], may be associated with reductions in suicidal behaviour [47]. Investigating the extent to which such activities in local populations have been, or can be, implemented in high risk areas, is important in increasing the effectiveness of suicide prevention programs. The impact of climate [48,49], socioeconomic factors [50-52] and natural disasters [53,54] on suicide should also be addressed in future research, especially in identifying the variation of these impacts across different areas.

## Conclusion

This study has described the spatial variation of suicide and clusters of high risk in Australia. The spatial and cluster analysis methods may have significant applications in mental health research and the development of effective suicide control and prevention strategies, especially after studying suicide patterns in more recent years. Exploring the spatiotemporal association between socio-environmental variables and suicide may also provide a better understanding of suicide cases at a local level and offer area-specific information to plan and implement suicide prevention activities.

## Competing interests

We declare that we have no competing interests.

## Authors’ contributions

XQ designed the study, implemented all statistical analyses and drafted the manuscript. ST conceptualised the idea and revised the study protocol, especially the research design and data analysis. WH provided advice on statistical analyses and interpretation of the results. AP helped interpret the results and assisted in drafting the manuscript. All the authors contributed to the preparation of the final manuscript and approved the submission.
